# Lung ultrasound training: a systematic review of published literature in clinical lung ultrasound training

**DOI:** 10.1186/s13089-018-0103-6

**Published:** 2018-09-03

**Authors:** Pia Iben Pietersen, Kristian Rørbæk Madsen, Ole Graumann, Lars Konge, Bjørn Ulrik Nielsen, Christian Borbjerg Laursen

**Affiliations:** 1 Department of Respiratory Medicine, Odense University Hospital, Sdr. Boulevard 29, 5000 Odense C, Denmark; 2 Institute for Clinical Research, University of Southern Denmark, Odense, Denmark; 3 Regional Center for Technical Simulation, Region of Southern Denmark, Odense, Denmark; 4 Department of Anaesthesiology and Intensive Care Medicine, Odense University Hospital, Odense, Denmark; 5 Department of Radiology, Odense University Hospital, Odense, Denmark; 6 Copenhagen Academy for Medical Education and Simulation (CAMES), University of Copenhagen and the Capital Region of Denmark, Copenhagen, Denmark

**Keywords:** Thoracic ultrasound, Pulmonary ultrasound, Point-of-care ultrasound, Medical education, Training

## Abstract

**Background:**

Clinical lung ultrasound examinations are widely used in the primary assessment or monitoring of patients with dyspnoea or respiratory failure. Despite being increasingly implemented, there is no international consensus on education, assessment of competencies, and certification. Today, training is usually based on the concept of mastery learning, but is often unstructured and limited by bustle in a clinical daily life. The aim of the systematic review is to provide an overview of published learning studies in clinical lung ultrasound, and to collect evidence for future recommendations in lung ultrasound education and certification.

**Methods:**

According to PRISMA guidelines, three databases (PubMed, Embase, Cochrane Library) were searched, and two reviewers examined the results for eligibility. Included publications were described and assessed for level of evidence and risk of bias according to guidelines from Oxford Centre for Evidence-Based Medicine and Cochrane Collaboration Tool for Risk of Bias assessment.

**Results:**

Of 7796 studies screened, 16 studies were included. Twelve pre- and post-test studies, three descriptive studies and one randomized controlled trial were identified. Seven studies included web-based or online modalities, while remaining used didactic or classroom-based lectures. Twelve (75%) studies provided hands-on sessions, and of these, 11 assessed participants’ hands-on skills. None of the studies used validated neither written nor practical assessment. The highest level of evidence score was 2 (*n* = 1), remaining scored 4 (*n* = 15). Risk of bias was assessed high in 11 of 16 studies (68.75%).

**Conclusion:**

All educational methods proved increased theoretical and practical knowledge obtained at the ultrasound courses, but the included studies were substantial heterogeneous in setup, learning-, and assessment methods, and outcome measures. On behalf of current published studies, it was not possible to construct clear guidelines for the future education and certification in clinical lung ultrasound, but the use of different hands-on training facilities tends to contribute to different aspects of the learning process. This systematic review proves a lack of learning studies within this content, and research with validated theoretical and practical tests for assessment is desired.

## Introduction

The clinical use of lung ultrasound (LUS) in emergency departments, critical care units as well as in respiratory departments has increased substantially. LUS has an excellent diagnostic accuracy for many of the most common causes of acute respiratory failure (e.g., cardiogenic pulmonary edema, pneumonia, pleural effusion, and pneumothorax) and increases the proportion of patients receiving a correct diagnosis and treatment [1–6]. Furthermore, LUS is a rapid, bedside, non-invasive, radiation-free diagnostic tool, which the clinician can use as an integrated part of the initial clinical assessment as well as for monitoring purposes. However, the value of LUS is dependent on competent operators performing the examination.

Several societies, e.g., the European Federation of Societies for Ultrasound in Medicine and Biology, British Thoracic Society and European Association of Cardiovascular Imaging, have clear guidelines and descriptions of logbook, number of performed supervised examinations needed, and basic knowledge curricula, which must be obtained before performing unsupervised lung ultrasound examinations [7–9]. However, no clear evidence-based guidelines or recommendations exist on the training needed to obtain adequate skills for performing an LUS examination.

Like other procedures and treatments, LUS education and certification should be based on best available evidence, and with gathered validity evidence in learning- or clinical studies. The aims of this systemic review were to provide an overview of the literature published in learning studies in clinical LUS, and to explore and collect evidence for future recommendations in lung ultrasound education and competency assessment.

## Materials and methods

The systematic review was performed according to the Preferred Reporting Items for Systematic Review and Meta-Analysis (PRISMA) guidelines [10]. A systematic literature search was conducted in PubMed, Embase, and Cochrane Library in collaboration with a research librarian from the Medical Research library at Odense University Hospital, Denmark. Terms used: lung OR lungs OR pulmonal OR pulmonary OR thoracic OR thorax OR thoracal OR mediastinal OR mediastinum, ultrasound OR ultrasonic OR ultrasonography OR ultrasonics OR sonography OR sonographic, medical education OR education OR learning OR training OR clinical competences OR curriculum including MeSH terms. The search was completed on March 7, 2017. The inclusion criterion was: learning- or education studies in lung or thoracic ultrasound. No exclusion criteria were provided within languages, animal studies, etc.

After removing duplicates, all titles and abstracts were screened by two authors (PP and KRM). All articles that potentially met the broad inclusion criterion or indeterminate articles were assessed with full article reading. Abstracts regarding the following studies were excluded: ultrasound education in other organ systems or anatomical structures than lungs or thorax, cost–benefit analysis, case reports, author responses, letter to the editor, and comments. Diagnostic accuracy studies were excluded from this review, except from those, which also included a learning study or had objectives or outcomes that assessed training or development of competencies in LUS. The same two authors then subsequently read all eligible articles, and each article was discussed until consensus. In case of disagreement, a third reviewer (CBL) was conferred. Hand search was conducted on references of included full articles. Level of evidence was categorized using the Oxford Centre for Evidence-Based Medicine (OCEBM) system for Level of Evidence [11]. Bias in each included article were discussed and marked according to Cochrane Collaboration risk of bias [12].

## Results

### Search strategy

**Fig. 1:**
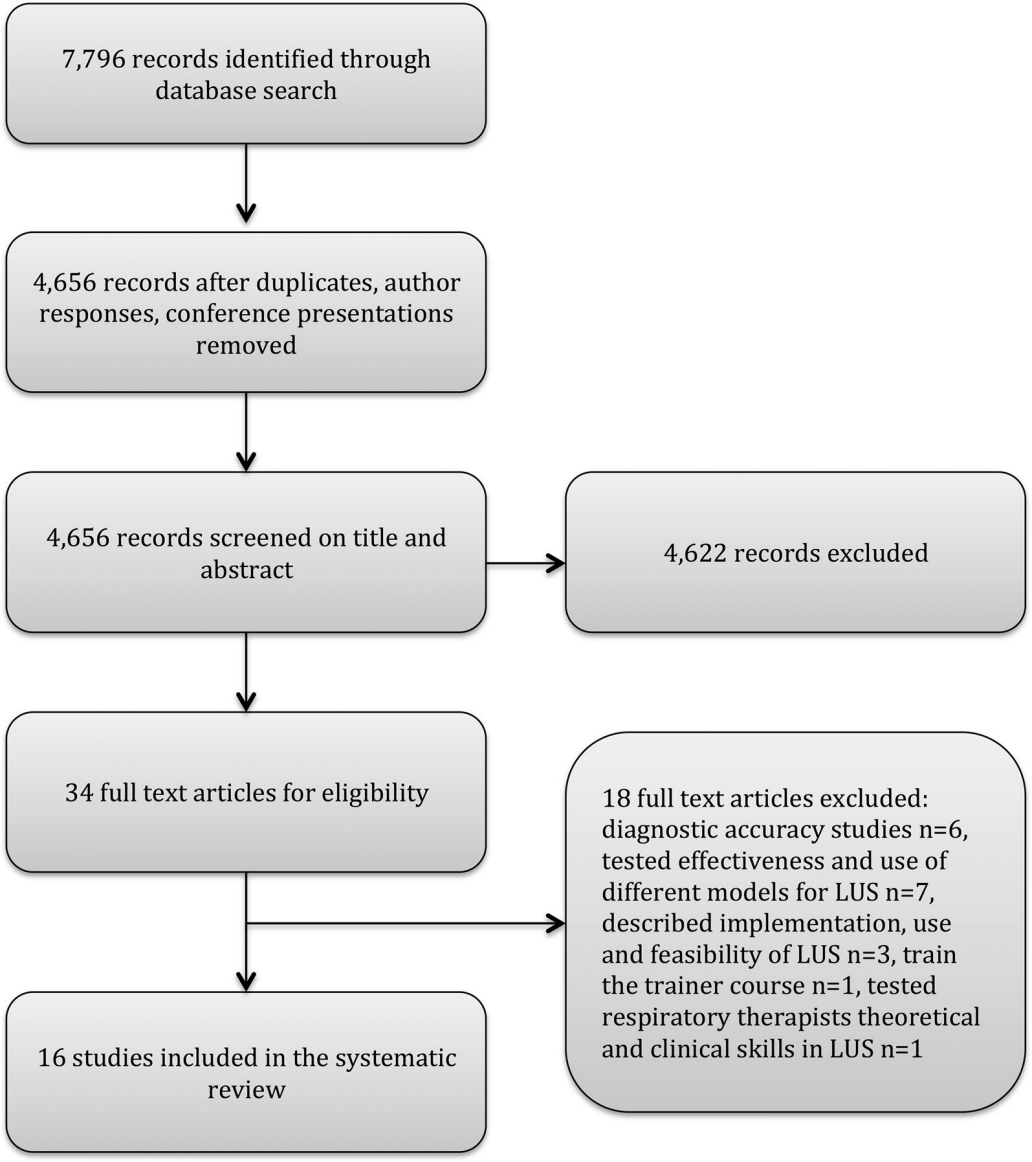
Flowchart of search strategy, and selection process based on the Preferred Reporting Items for Systematic Reviews and Meta-analysis (PRISMA)

The initial search yielded 7796 publications. After removal of duplicates, author responses and conference abstracts, 4656 publications remained. Of these, 4622 were excluded. Most of the excluded studies did not meet the inclusion criterion at all, and comprised complete different topics, aims, and objectives than education or assessment in LUS or thoracic ultrasound. Because of the wide search strategy, the amount of publications not relevant for this systematic review was large. Figure 1 presents the eligibility process and exclusion of articles. Causes of the full-text exclusions were: diagnostic accuracy studies (*n* = 6), testing the effectiveness and use of different models/phantoms or hands-on facilities for LUS (*n* = 7), describing implementation, use and feasibility of LUS (*n* = 3), train-the-trainer course (*n* = 1), and assessment of respiratory therapists’ theoretical and clinical skills in LUS (*n* = 1). The reference lists of included papers were screened without leading to inclusion of further studies. Study design, participants, learning strategy, hands-on facilities, and assessment are described below. Additional information is shown in Tables 1 and 2.

**Table 1:** Publications on education in lung ultrasound: study characteristics

	Study design	Assessment	Facility	Education tool	Participants
Pre and post-test studies					
Noble et al. [13]	Pre- and post-test	Theoretical pre- and post-test (50 video clips)	Ultrasound video clips	2 h didactic lecture (one for pneumothorax and one for pulmonary oedema)	27 physicians working for the SAMU at l’Hôpital Necker in Paris, France
Oveland et al. [14]	Pre- and post-test and 6-months follow-up	Theoretical pre- and post-test (34 MCQs, 10 US physics questions, 17 recognition pictures, 7 video clips) and hands-on practical examination (6 months follow-up	Healthy live models and porcine models	8 h attendance course including didactic (2 h), practical (2 h) and experimental (4 h) sessions	20 first-year to graduate-year medical students (11 at 6 months follow-up)
Breitkreutz et al. [15]	Pre- and post-test	Theoretical pre- and post-test (15 MCQs and 5 recognition images), post course recognition quiz (15 video clips) and practical post course examination (16 predefined sonoanatomic items)	Healthy live models, patients with chronic or malignant lung diseases or who had recently underwent thoracotomy. Custom-made gel phantoms	Two and a half hour theoretical training (six brief lectures in anatomy, physiology and pathology of thorax and four case presentations). Two and a half hour hands-on training	54 trainees. Group A: 14 medical students, Group B: 32 anaesthesiologists, Group C: 8 trauma surgeons
Cuca et al. [16]	Pre- and post-test	Theoretical pre-, post-, and sustainability test (20 MCQs, results compared with THOLUUSE-study [14])	–	E-learning module including physiological and pathological sonographic patterns (Five topics: basics and pleural effusion, pneumothorax, pulmonary oedema and consolidation, trachea, workflow of LUS) estimated time 30–50 min	29 medical students and medical doctors
Hulett et al. [17]	Pre- and post-test	Theoretical pre- and post-test (46 questions including MCQs, true/false, matching items, fill-in-the blank image and video recognition), practical pre- and post course skills	Patients located in medical ICU at the North Carolina Hospital	1 h didactic instruction, 1 h image interpretation workshop and image acquisition training, in 1 work week supervised hands-on training	Eight critical care medicine fellows
Bhat et al. [18]	Pre- and post-test and 1-week follow-up	Theoretical (16 MCQs)	Ultrasound images and video clips obtained in the Emergency Department by trained ultrasound physicians	1 h didactic lecture including basic scan technique, normal ultrasound anatomy, image interpretation of normal or pathological pattern	57 prehospital providers (19 medical technicians students, 16 paramedic students, 18 certified medical technicians and four certified paramedics
Connolly et al. [19]	Pre- and post-test	Theoretical pre- and post-test (21 MCQs) and practical examination (real-time scans saved and blinded evaluated by instructors)	Live models and phantom task trainer models and simulators	Five 1-h workshops and 4 h didactic online preparatory training. Hands-on with supervised scans	24 medical students in MCQ pre- and post-test evaluation and 16 in clinical skill assessment
Dinh et al. [20]	Pre- and post-test and 3 months follow-up	Theoretical pre-, post-, and sustainability test (50 MCQs—12 pulmonary) (84 point checklist). Pathologic image interpretation (4 cases with each 20 questions each). Ultrasound comfort level and use of ultrasound	Healthy live models, simulators	2 days course including didactic lectures, live demonstrations, hands-on sessions on healthy models, pathologic image interpretation with cases using ultrasound simulator	Eight ICU fellows, participants with the previous ultrasound experience were excluded
Heiberg et al. [21]	Pre- and post-test	Theoretical pre- and post-test (56 MCQs) and practical three test sessions	Four healthy medical students	E-learning course including text, pictures, animations and movies (5–8 h) and hands-on session (4 h; 30 min LUS)	20 medical students
Sanchez-de-Toledo et al. [22]	Pre- and post-test	Theoretical pre-, and post-test (four written cases). Practical skill test after 60 min hands-on session (four cases)	Porcine models	One and a half hour theoretical and practice-based course	Four veterinaries, eight neonatologists, seven paediatric intensive care specialists, two intensive care nurses, three paediatric surgeons, eight paediatric anaesthesiologists, four paediatricians
See et al. [23]	Pre- and post-test	Theoretical pre- and post-test (20 MCQs) and hands-on (blinded evaluation of image acquisition and interpretation)	Mechanically ventilated patients or patients with respiratory failure, requiring at least 40% inspired oxygen fraction to maintain an oxygen saturation of 90%	30 min didactic introduction, 1 month self-study (powerpoint slides, criticalecho.com and court.net). Supervised scans with immediately feedback with focus on image acquisition, afterwards image interpretation by blinded observer	22 respiratory therapists
Greenstein et al. [24]	Pre- and post-test	Theoretical pre- and post-test (20 MCQs) and hands-on assessment	Healthy human models	3 days course including didactic lectures with real-time ultrasound scan on healthy models, image interpretation sessions and hands-on training	363 critical care physicians, hospitalists, surgeons, physician assistants, advanced practice nurses and medical residents
Descriptive studies					
Krishnan et al. [25]	Post course evaluation and sustainability test	Theoretical post- and sustainability test (20 video clips ± pneumothorax)	Ultrasound video of 53 patients before and after elective thoracic surgery. In all, 99 videos were compiled (52 without pneumothoraces and 47 with)	5-min online presentation of the use of ultrasound for detection of pneumothorax	79 (70 at 6 month follow-up) residents and faculty members from Department of anaesthesia
Abbasi et al. [26]	Prospective cross-sectional study	Hands-on assessment (± pneumothorax)	Healthy live models and patients admitted in Emergency Department with thoracic trauma	2 h training course including 30 min didactic lecture, 30 min hands-on training on healthy volunteers, 1 h training on patients	Four emergency physicians
Gargani et al. [27]	Post course evaluation	Online assessment of uploaded LUS examinations and theoretical assessment of *b*-line interpretation (44 videos)	Patients	Part A: web-based training program; 26 min educational video with focus on *b*-line assessment. Upload of 7 self-performed lung ultrasound videos, when videos were approved by experts, trainees proceed to Part B: *b*-line interpretation	Thirty nephrologists and 14 cardiologists
Randomized controlled trial					
Edrich et al. [28]	Randomized controlled trial with 4 weeks follow-up	Theoretical pre-, post-, and sustainability test (10 MCQs and one video clip) and practical examination (blinded reviewers)	Healthy live models	Group I: web-based (powerpoint 25 min and online demonstration 5 min). Group II: Classroom-based (powerpoint) 45 min didactic lectures and 20 min hands-on training. Group III: No education or hands-on training. Blinded reviewers	138 anaesthesiologists from four academic hospitals. Participants with the previous ultrasound experience excluded

**Table 2:** Publications in education in lung ultrasound: study statistics and conclusion

	Statistical analysis	Outcome measures	Study conclusion	Level of evidence
Noble et al. [13]. Evaluation of thoracic ultrasound training module for the detection of pneumothorax and pulmonary edema by prehospital physician care providers. 2009	Paired *t* test compared mean score of pre- and post-test	Improvement in pre- and post-test scores	With minimal didactic and image recognition skill sessions are needed before physicians can recognize the key artifacts, which lead to the diagnosis of pulmonary edema and pneumothorax.	4
Oveland et al. [14]. Animal laboratory training improves lung ultrasound proficiency and speed. 2013	Sensitivity, specificity, positive and negative predictive value	Confidence level, scan time, improvement in theoretical score and sensitivity/specificity	Novices can quickly learn how to diagnose PTX using lung US. Training in an animal facility imparts a high level of long-term diagnostic proficiency and speed for diagnosing PTX	4
Breitkreutz et al. [15]. Thorax, trachea and lung ultrasonography in Emergency and Critical care medicine: Assessment of an Objective Structured training concept. 2013	Non-parametric Wilcoxon matched pairs (within groups), Mann–Whitney *U* test (between groups)	Improvement in pre- and post-test scores. Recognition and interpretation skill scores. Practical imaging performance scores	1-day training program like THOLUUSE significantly improves theoretical and practical skills for sonographic diagnosis of including PLE and PTX	4
Cuca et al. [16]. Assessment of a new e-learning system on thorax, trachea and lung ultrasound. 2013	Wilcoxon matched pairs test. Self-assessment survey	Improvement in pre- and post-test score and sustainability test, qualitative program evaluation score	Results of written tests from the e-learning attendance course are comparable and with same progress as attendance-based courses	4
Hulett et al. [17]. Development and Preliminary Assessment of Critical Care Ultrasound Course in an Adult Pulmonary and Critical Care Fellowship Program. 2014	Paired t-test on pre- and postcourse performances	Improvement in pre- and post-test scores, practical pre- and postcourse skill score and self-assessment score	A formal curriculum dedicated to critical care ultrasound can be developed and implemented on site in a fellowship training program. After validation studies testing longer term retention of knowledge and bedside skills on trainees at other broadly representative medical centres, the curriculum described here might form the basis of a widely applicable onsite critical care ultrasound course curriculum	4
Bhat et al. [18]. Prehospital Evaluation of Effusion, Pneumothorax and standstill (PEEPS): Point-of-care Ultrasound in emergency medical services. 2015	Two-tailed, paired *t* test	Improvement in pre-, post- and sustainability test. Level of confidence	This study showed potential promise for training prehospital EMS providers in accurate US interpretation through a 1-h didactic lecture focused on US technique and anatomy for the assessment of pericardial effusion, pneumothorax, and cardiac standstill	4
Connolly et al. [19]. Ultrafest: a novel Approach to Ultrasound in Medical Education Leads to Improvement in Written and Clinical Examinations, 2014	Paired *t* test analysis	Improvement in pre- and post-test score and practical pre- and postcourse skill score	A 1-day, 9-h, small group instruction and practice symposium improved student knowledge on trauma and pulmonary US, and improved image acquisition, but the latter fell short of significant proficiency	4
Dinh et al. [20]. Impact of a 2-day critical care ultrasound course during fellowship training: a pilot study. 2015	Students *t* test, Chi square or ANOVA	Improvement in pre-, post-, and 3 month follow-up test score, comfort level score. Number of self-reported scans	Introduction of a 2-day critical care ultrasound course has both a positive short- and long-term impact on fellows’ confidence and proficiency with ultrasound use. Utilizing tools such as written tests to assess basic knowledge, live models to teach practical skills, and ultrasound simulators to teach pathological image identification can help standardize critical care ultrasound training	4
Heiberg et al. [21]. Point-of-care clinical ultrasound for medical students. 2015	Paired Students *t* test, Wilcoxon rank sum test, Chi squared test, linear regression	Improvement in pre- and post-test score and practical pre- and postcourse skill score	Medical students with no previous experience of ultrasound techniques demonstrated a significant increase in their ability to acquire and interpret an ultrasound image after completion of interactive e-learning, and this competence was further improved after 4 h of systematic hands-on training	4
Sanchez-de-Toledo et al. [22]. Teaching chest ultrasound in an porcine model. 2016	Sensitivity, specificity, positive and negative predictive values	Improvement in sensitivity and specificity after 30 and 60 min	Brief training in theory combined with animal models facilitates learning for medical professionals with no previous training in US and enables them to recognize the three most relevant thoracic US patterns. The introduction of advanced simulation with animal models can facilitate training of personnel in the recognition and management of acute lung disease	4
See et al. [23]. Lung ultrasound training: curriculum implementation and learning trajectory among respiratory therapists. 2016	Paired *t* test. Three patients block (36 images) Overall performance score with linear regression	Improvement in pre- and post-test scores and practical skill scores	We devised a pragmatic lung ultrasound curriculum, which involved building rapport, stimulating self-directed learning, and avoiding cognitive overload. Our training method allowed RTs to acquire the ability to independently perform lung ultrasound after at least ten directly supervised scans	4
Greenstein et al. [24]. Effectiveness of a Critical Care ultrasonography Course. 2016	Two-tailed student *t* test	Improvement in pre- and post-test score and practical pre- and postcourse skill score	This 3-day CHEST CCUS course is an effective method to train large groups of clinicians in the skills requisite for CCUS. The majority of learners demonstrated improved performance in both image interpretation and hands-on ultrasonography skills across all educational domains at the completion of the course	4
Krishnan et al. [25]. Efficacy of an online education program for ultrasound diagnosis of pneumothorax. 2013	Sensitivity and specificity at the time of educational program and after 6 months	Improvement in sensitivity/specificity, use of ultrasound from baseline to follow-up	After viewing a 5-min online training video, physicians can reliably rule out pneumothorax on an optimal ultrasound image. They are also able to retain this skill for up to 6 months	4
Abbasi et al. [26]. Accuracy of emergency physician-performed ultrasound in detecting traumatic pneumothorax after 2-h training course. 2012	Sensitivity and specificity, positive after 5, 10 and 20 ultrasound examinations, × 2-test for proportions and the Student *t* test for continuous variables	Improvement in sensitivity/specificity after 5, 10 and 20 examinations	By a brief learning course, the emergency physicians easily diagnosed PTX in trauma patients with a reasonable accuracy in comparison with CT scan as the gold standard	4
Gargani et al. [27]. Efficacy of a remote web-based lung ultrasound training for nephrologists and cardiologists: an LUST trial sub-project. 2016	Mean number of *b* lines ± SD. Pearsons correlation coefficient (trainer vs. trainee). Intraclass correlation coefficient and confidence interval. Interobserver agreement by Bland–Altman plot	Test-score agreement (trainee vs. trainer)	In conclusion, this study performed in the framework of the LUST trial shows that nephrologists and cardiologists can be effectively trained to measure lung congestion by an entirely web-based educational program	4
Edrich et al. [28]. A comparison of web-based with traditional classroom-based training of lung ultrasound for the exclusion of pneumothorax. 2016	Agreement of reviewers results assessed with Krippendorff test. Total score in percent. One-sided, 2-sample *t* test	Improvement in pre-, post-, and sustainability test score and practical test	When training anaesthesiologists to perform LUS for the exclusion of pneumothorax, we found that web-based training was not inferior to traditional classroom-based training and was effective, leading to test scores that were similar to a group of clinicians experienced in LUS	2

### Study design

In total, there were 12 pre- and post-test studies that used improvement in written test scores to evaluate the educational Cochrane [13–24]. Five of the pre- and post-test studies had a follow-up time from 1 week to 6 months, average 13 weeks ± 4.83 [14, 16, 18, 20, 25], and one recorded number of scans performed from baseline to follow-up [20]. Three descriptive studies were identified [25–27] and one randomized controlled trial [28]. Five of the studies (31%) were courses in general critical care ultrasound, or basic skill ultrasound, where thoracic or lung ultrasound was a specific and independently evaluated topic [17, 19–21, 24].

### Participants

Most study participants were ultrasound novices, and especially novices in clinical LUS, and varied from medical students to respiratory therapists, emergency department residents, and anesthesiologists. Three studies also included other healthcare professionals as prehospital providers, nurses, and veterinarians [18, 22, 24]. Two studies excluded participants with the previous ultrasound certification or attendance in a formal critical care ultrasound course within 12 months [20, 28], and two studies only included a study population with no experience [21, 24].

### Learning strategy

Learning strategies in the studies included were heterogeneous in both time spent on lectures, theoretical presentation, and method used for assessment. The most commonly used educational tool used was didactic lectures (*n* = 12, 75%), with a variation of time spent from 30 min sessions [26] to 2.5 h sessions [15]. Abbasi et al. presented a single topic course (detection of pneumothorax with LUS), and time spent on didactic lecture was 30 min. This study was the only single topic course that used didactic lecture as educational tool [26]. Remaining studies introduced classroom-based learning covering a more comprehensive introduction to full LUS, primarily with 15–30 min education in each of the main topic. Some studies had a clear overview and description of topics included in the didactic lectures, whereas other studies only stated the overall general topics (Table 1).

Four studies describe a full day to 3 days courses with alternating theoretical and hands-on sessions [14, 19, 20, 24]. Four studies incorporated live ultrasound examinations by instructors in the theoretic session to combine the theoretic and practical understanding [19, 20, 24, 26]; otherwise, images and video clips were frequently used in the lectures.

Web-based learning or online presentations were used in 7 (44%) studies [16, 19, 21, 23, 25, 27, 28]. Four of those had only online presentations or web-based learning modules without didactic lectures or hands-on sessions [16, 25, 27, 28]. Cuca et al. studied a web-based learning program evaluated by nine experts of the international lung ultrasound consensus committee [16], and used the same written tests, topics, and curriculum as the study by Breitkreutz et al. [15]. Cuca et al. compared the results from the two studies. Krishnan et al. [25] presented a 5 min online presentation in the use of ultrasound as a diagnostic tool to confirm pneumothorax. Gargani et al. had a 26 min online presentation with primary focus on *b*-line presentation, interpretation, and the possibility of real-time demonstrations or meeting with instructors on Skype. Subsequently, participants were to upload seven LUS examinations for evaluation. When the instructors had approved the seven videos, the participants could proceed to the second part of the training, including a set of 44 videos with the focus of counting *b* lines [27]. In the randomized trial by Edrich et al., one of the study groups received a web-based educational learning program and had no hands-on session, another group had a 45 min classroom-based lecture and 20 min hands-on, whereas the control group had no lectures at all. The participants were evaluated with a pretest, post-test, and 4 week retention test [28].

### Hands-on training facilities

Twelve of sixteen studies included hands-on sessions in the educational program [13–15, 17, 19–24, 26, 28]. Simulators were used in three studies [19, 20, 26], and healthy live models in eight studies [14, 15, 19–21, 24, 26, 28]. In five studies, emergency department patients or patients with respiratory failure in other departments were assessed as a part of the training program [15, 17, 23, 26, 27], including three studies, where LUS video clips from patients hospitalized were obtained and used in the assessment [13, 18, 25]. Porcine models were used in two studies [14, 22]. Four studies combined the use of different models, patients and/or simulators [14, 15, 19, 20, 26].

### Assessment

Thirteen studies used written examinations to assess theoretical knowledge obtained at the educational programs [13–25]. They all used multiple-choice items format covering true/false questions, one-best-answer questions, single-correct-answer questions and multiple-response questions, all included images and/or video clips in the questions. None of the studies described gathering validity evidence for neither the pre- and post-tests nor the practical skill assessment tools. One study, however, had the multiple-choice questions (MCQs) peer-reviewed by the instructors ahead of the study [20], but the vast majority of the assessment checklists, written tests, and curricula were described as based on the international consensus recommendations for point-of-care lung ultrasound by Volpicelli et al. [29].

Eleven studies assessed participants’ practical skills [14, 15, 17, 19–24, 26, 28]. The most common method used for evaluation and assessment of practical skills was observer checklists but varied greatly. Participants in See et al. [23] scanned 12 zones with an instructor bedside, who was allowed to comment or help if needed, videos were stored, and participants then interpreted the clips in front of the instructor. Connolly et al. [19] assessed the participants’ practical skills by letting participants scan four windows, and videos were stored and rated by blinded instructors. Breitkreutz et al. [15] had 16 predefined sonoanatomical structures that participants should present and were then rated on a standardized sheet. Respectively, 46 and 84 checklist items were to be scanned in Hulett et al. and Dinh et al. [17, 20] and were evaluated regarding image acquisition and interpretation. Furthermore, Dinh et al. presented four cases with 20 case questions each [20]. Heiberg et al. [21] performed online testing of the students’ practical skills by correct/incorrect and offline evaluation of image quality and interpretation. Greenstein et al. used 20 standardized examination tasks and 20 video-based examinations [24], whereas Oveland et al. presented scans on porcine models with confirmation or validation of pneumothorax, oral feedback from instructor and yet another scan session [14].

Level of evidence of the included studies is presented in Table 2 according to OCEBM guidelines, and assessment of risk of bias in Table 3. No studies scored the highest level of evidence, one study scored 2, remaining part of the studies scored 4. Bias was assessed as high in the majority of the studies (Table 3).

**Table 3:** Scores of the Cochrane Collaboration risk of bias assessment tool [12]

	Selection bias		Performance bias	Detection bias	Attrition bias	Reporting bias	Other bias	Overall risk of bias
	Random sequence generation	Allocation concealment	Blinding of participants and personnel	Blinding of outcome assessment	Incomplete outcome data	Selective reporting	Other sources of bias	
Pre and post-test studies								
Noble et al. [13]. 2009	#	#	1	1	1	1	?	Low
Oveland et al. [14]. 2013	#	#	?	0	0	0	?	High
Breitkreutz et al. [15]. 2013	#	#	1	0	1	1	?	Low
Cuca et al. [16]. 2013	#	#	0	1	0	1	?	High
Hulett et al. [17]. 2014	#	#	0	0	1	0	?	High
Bhat et al. [18]. 2015	#	#	0	0	0	1	?	High
Connolly et al. [19]. 2014	#	#	1	0	0	0	?	High
Dinh et al. [20]. 2015	#	#	0	0	1	1	?	High
Heiberg et al. [21]. 2015	#	#	0	0	1	0	?	High
Sanchez-de-Toledo et al. [22]. 2016	#	#	1	1	1	1	?	Low
See et al. [23]. 2016	#	#	0	0	1	1	?	High
Greenstein et al. [24]. 2016	#	#	1	0	0	0	?	High
Descriptive studies								
Krishnan et al. [25]. 2013	#	#	0	1	1	1	?	Low
Abbasi et al. [26]. 2012	#	#	0	0	1	1	?	High
Gargani et al. [27]. 2016	#	#	0	0	1	1	?	High
Randomized controlled trial								
Edrich et al. [28]. 2016	?	0	1	1	1	1	?	Low

0 = high risk of bias, 1 = low risk of bias, ? = unclear risk of bias, # = irrelevant in this study (non-randomized trial)

## Discussion

The vast majority of the currently published LUS learning studies are one-group pre- and post-tests studies with low level of evidence. This study design can just inform us that trainees learned something from the specific intervention, but does not provide any evidence on how to build a curriculum [30]. The studies are heterogeneous in choice of: educational program, teaching methods, participant assessment, and study outcome. In addition to conventional classroom-based didactic lectures, web-based learning was often chosen as an alternative or additional method and was used in 7 of the 16 included studies [16, 19, 21, 23, 25, 27, 28], but only one study measured the effect of the two educational methods, and compared the results from the two groups in a randomized controlled trial [28].

Web-based learning strategies have been proven to have several advantages. Ruiz et al. describe increased accessibility and flexibility as important advantages. It standardizes course content and delivery independent of teacher presentation and variation. Students are in control of their learning sequence and learning pace, and web-based learning can be designed to include outcome assessment [31, 32]. Furthermore, it is possible to implement different types of multimedia such as graphics, videos, animations, and texts to increase learning ability. A meta-analysis by Cook et al. [33] proved that medical web-based learning was significantly superior to no intervention, and participants could achieve results similar to traditional learning methods like classroom-based learning in numerous diagnostic and therapeutic content areas. Edrich et al. [28] correspondingly found the same improvement. Since web-based education has similar outcome as classroom-based lectures, it would be obvious to include other parameters like maintenance of both theoretical and practical skills with follow-up assessments, time efficiency, and user satisfaction surveys. The meta-analysis, like this systematic review, suffers from considerable heterogeneity in study participants, learning methods, and outcome measures.

Web-based learning in general point-of-care ultrasound has advantageously been evaluated in several studies [34–36]. In Kang et al. [36], outcome measures were not only improvement in test score, but also hours spent on organizing the course and course costs. In both cases, web-based learning was more cost-effective. None of the studies included in this systematic review incorporated cost–benefit analysis, but one concluded that an ultrasound symposium requires a massive setup and great financial resources because of the number of ultrasound machines, phantoms, volunteers, instructors, and rooms. When building a theoretical curriculum in medical education, the teacher:student ratio can be low without affecting the learning ability significantly. However, when training practical skills, it requires a closer relation and interaction between instructor and trainee, and the most optimal trainee to instructor ratio is as close as 1:1 as possible. Oveland et al. [14] also discussed cost–benefit issues and concluded that porcine models as simulators and animal laboratory training in general, combined with ethical considerations, may be an option but have time, venue, and cost dilemmas.

The practical skill assessments of course participants in the included studies diverge in amount of checkpoints and topics. Even though the studies included used various checklists to keep the assessment as objective and standardized as possible, only two studies had blinded reviewers scoring the stored images or ultrasound sequences afterwards [19, 28], and no validity evidence was provided for any checklists.

LUS imaging and examinations differ from other point-of-care ultrasound examinations, because image interpretation and pathological recognition are based on sonographic artifacts instead of directly imaging diagnostics as, e.g., thickening of gallbladder wall, pericholecystic fluid, and sludge as a sign of acute cholecystitis. Therefore, there is a great need for a standardized and validated tool for assessing the understanding of LUS, image acquisition, and image interpretation, additionally, to demonstrate the capability to correlate the patterns and interpretations to lung pathology and physiology.

In general, when introducing a new assessment tool, validity evidence should be gathered, to ensure the reliability, and to make it possible for meaningful interpretation. Today, one of the most described and recognized frameworks for validity testing is by Messick [37]. Five distinct sources of validity evidence in scientific experimental data have been discussed; content, response process, internal structure, relationship to other variables, and consequences [38]. Some types of assessment demand a stronger emphasis on one or more sources of evidence depending on the curriculum, consequences, and properties of inferences. All sources should be researched with the highest level of evidence possible, but within this setting, an assessment tool should emphasize content-related evidence with some evidence of response quality, internal structure, and consequences.

A new study have constructed and gathered validity evidence for an instrument to assess LUS competences by obtaining international consensus by experts in multiple specialties [39]. The objective structured assessment of lung ultrasound skills (LUS-OSAUS) could form the foundation of further and more homogeneous studies in the future.

The theoretical assessment was a preferred method for measuring the degree of obtained theoretical knowledge before and after a course, but single-group pretest post-test design suffers from minimal internal and external validity. In the case of evaluating medical education through this set-up, it would be surprising if an increased post-test score was not found. This setup has been discussed and criticized for decades and is today considered obsolete [30, 40, 41]. A single topic curriculum like presented in Krishnan et al., where participants were presented for a 5 min online presentation in detection of pneumothorax with LUS, and assessed theoretical with 20 videos, proves that even a very short theoretical session leads to increased knowledge and pattern recognition. However, it does not provide any guarantee that the trainees can obtain the ultrasound images themselves, or connect the patterns to relevant differential diagnosis in a clinical setting.

One study reported that their theoretical test was validated, but did not describe how this was done [18]. Another had the questions peer reviewed by authors of the study [20]. Written tests, in general, are proven to be authoritative motivating, facilitating the learning process and cost-effective [42]. Disadvantages of using the same theoretical test as pretest, post-test, and follow-up test are recall bias or “learning the test” [43, 44]. The majority of the studies have tried to eliminate this bias by changing the order of questions as well as the order of answers. None of the participants in the included studies were blinded to the studies. Since the participants knew that they were being evaluated, they may have been more motivated to enhance their performance in the tests.

There were large differences in the use of healthy live models, patients with respiratory failure or lung diseases, phantoms/simulators, or porcine models for the hands-on training. The overall conclusion was that all models could contribute to increased hands-on competencies. Summarized, the different models could contribute to different aspects of the learning process; healthy live models were well suited for getting comfortable with the ultrasound devices, learning advantages and disadvantages of various transducers, improving image optimization, and learning hand–eye coordination. When using porcine models, it was possible to create pneumothoraces or pleural effusions allowing trainees to train the visual understanding of these diagnoses, but as discussed animal laboratory models have several other limitations. Dinh et al. [20] discuss the use of patients in an educational setting, and found it difficult to incorporate and standardize live pathology given the logistical challenges of recruiting patients with specific diseases and sonographic pattern. See et al. [23] reported problems with only a minority of the trainees scanned patients with pneumothorax due to a low prevalence of pneumothoraces. In addition, it is crucial not to delay diagnostic or initial treatment when using admitted patients in a learning study. Two studies used simulators for learning pathological patterns; both found simulators useful, and state that with the use of simulators, the students engage in both acquiring image and interpreting the abnormal finding while assimilating muscle memory with cognitive learning [20].

We acknowledge that the literature review was constrained by the quantity and quality of available evidence. Three databases were searched, decided being relevant for the topic, but a broader search strategy could potentially reveal more studies eligible for this systematic review, and we did not include data that were not published. However, all reference lists of publications eligible for full-text reading were searched with no additional findings. A minor part of the excluded publications contains education in lung ultrasound in context with ultrasound in other organ systems, e.g., abdominal ultrasound or eFAST (extended focused assessment with sonography for trauma). Different alternative expanded protocols for lung ultrasound or combined ultrasound have been developed and anchored in different specialties, and the evaluation of education of these different protocols was beyond the aim of this study. Therefore, studies were only included if the educational outcome was based on lung ultrasound separately.

The included studies failed to contribute to compelling body of evidence to support the educational evidence in LUS, and a meta-analysis was not possible to conduct because of the differences in assessment tools, and lack of comparability.

Standardized recommendations for education and certification in LUS is not possible to establish based on published studies because of heterogeneity in study design, low evidence-level, and high risk of bias among included literature. All courses showed progress in both theoretical and practical skills no matter which educational method used. If recommendations should be assigned from the current studies included in this systematic review and existing medical education literature, it would be ideal to use a three-step mastery-learning approach. First, trainees should obtain theoretical knowledge through either classroom-based education or web-based lectures with a curriculum based on experts’ opinion and a validated post-test with a pass–fail standard to ensure sufficient theoretical knowledge. Second, focused hands-on sessions on simulators, pigs, or healthy subjects until competency are demonstrated in the training environment using a performance test with solid evidence of validity. Third, supervised scanning of real patients with feedback from a trained instructor who preferably uses an assessment tool to decide when the trainee is ready for independent practice. Virtual-reality simulators could play an important role in the training of LUS, especially of pathologic cases, and could also provide standardized and objective assessments of competence. As far as we know, no studies have developed valid simulator-based tests of competence in LUS, even though simulators are commonly used in other specialties and are demonstrated to have a great potential for reproducible and objective assessment and effects on skill and behavior [45–47].

In conclusion, more uniform, competency-based training programs and assessment tools are needed to ensure a higher standard of education and assessment in LUS. Furthermore, simulation training could potentially `bute to the hands-on training in a calm environment making it possible to train high-risk cases without putting patients in risk.

## Abbreviations

LUS: lung ultrasound
BTS: British Thoracic Society
EFSUMB: European Federation of Societies for Ultrasound in Medicine and Biology
PRISMA: Preferred Reporting Items for Systematic Review and Meta-analysis
OCEBM: Oxford Centre for Evidence-Based Medicine
MCQ: multiple-choice question
